# Antihyperglycemic Effects and Molecular Mechanisms of *Ibervillea sonorae* (S. Watson) Greene: A Systematic Review of Preclinical Evidence

**DOI:** 10.3390/plants15172710

**Published:** 2026-09-03

**Authors:** Vanessa Wendi Suárez-Barrios, Ana Lilia Hernández-Alba, Juan Gabriel Juárez-Rojas, Abraham S. Arellano-Buendia

**Affiliations:** 1 Sección de Estudios de Posgrado e Investigación, Escuela Superior de Medicina, Instituto Politécnico Nacional, Mexico City 11340, Mexico; vsuarezb2400@alumno.ipn.mx (V.W.S.-B.); ahernandeza2312@alumno.ipn.mx (A.L.H.-A.); 2 Pharmacology Department, Instituto Nacional de Cardiología Ignacio Chávez, Mexico City 14080, Mexico; gabriel.juarez@cardiologia.org.mx; 3 Department of Cardio-Renal Pathophysiology, Instituto Nacional de Cardiología Ignacio Chávez, Mexico City 14080, Mexico

**Keywords:** *Ibervillea sonorae*, diabetes mellitus, cucurbitacins, glucose metabolism, medicinal plants, AMPK, AKT, systematic review

## Abstract

Diabetes mellitus is a major global health challenge, and medicinal plants represent an important source of bioactive compounds with therapeutic potential. *Ibervillea sonorae* (S. Watson) Greene is traditionally used in Mexican medicine for diabetes management and has attracted increasing scientific interest because of its reported antihyperglycemic activity. This systematic review critically evaluated the available preclinical evidence regarding the antihyperglycemic effects of *I. sonorae*, its proposed molecular mechanisms, phytochemical context, and methodological limitations. Following the PRISMA 2020 guidelines, PubMed, Scopus, and Google Scholar were searched for studies published between January 2010 and February 2026. Eight studies met the inclusion criteria, including animal, cellular, and enzyme-based models. The available evidence indicates that *I. sonorae* preparations may improve glycemic control through complementary mechanisms involving enhanced glucose uptake and insulin-related responses, including participation of the PI3K/AKT/GLUT4 signaling network, together with inhibition of α-glucosidase and α-amylase activity. AMPK-related mechanisms remain biologically plausible but have not been directly established across the included studies. Phytochemical evidence identifies cucurbitacin-derived constituents, including kinoins, as potentially relevant bioactive compounds; however, most mechanistic findings were obtained using crude extracts or other plant preparations, and their specific contribution cannot yet be attributed to individual constituents. Overall, this review integrates the fragmented preclinical evidence and identifies the principal mechanistic, phytochemical, and methodological gaps that must be addressed before clinical translation. *I. sonorae* represents a promising candidate for antihyperglycemic research, although phytochemical standardization, rigorous mechanistic studies, comprehensive safety evaluation, and well-designed clinical investigations are required to establish its therapeutic efficacy and safety.

## 1. Introduction

The global prevalence of diabetes continues to rise, representing a major public health challenge worldwide [1]. Diabetes mellitus is a chronic metabolic disorder characterized by persistent hyperglycemia resulting from defects in insulin secretion, insulin action, or both. Type 1 diabetes mellitus results from autoimmune destruction of pancreatic β-cells, whereas type 2 diabetes mellitus is primarily characterized by insulin resistance and progressive β-cell dysfunction [2]. Persistent hyperglycemia contributes to the development of severe microvascular and macrovascular complications, including nephropathy, neuropathy, retinopathy, and cardiovascular disease.

Despite the availability of multiple pharmacological therapies, diabetes remains a major cause of morbidity and mortality worldwide. Furthermore, adverse effects, treatment costs, and limited accessibility in some populations have encouraged the search for novel therapeutic alternatives. In this context, medicinal plants and their bioactive compounds have attracted considerable attention because of their potential antihyperglycemic, antioxidant, and anti-inflammatory properties [3].

**Figure 1:**
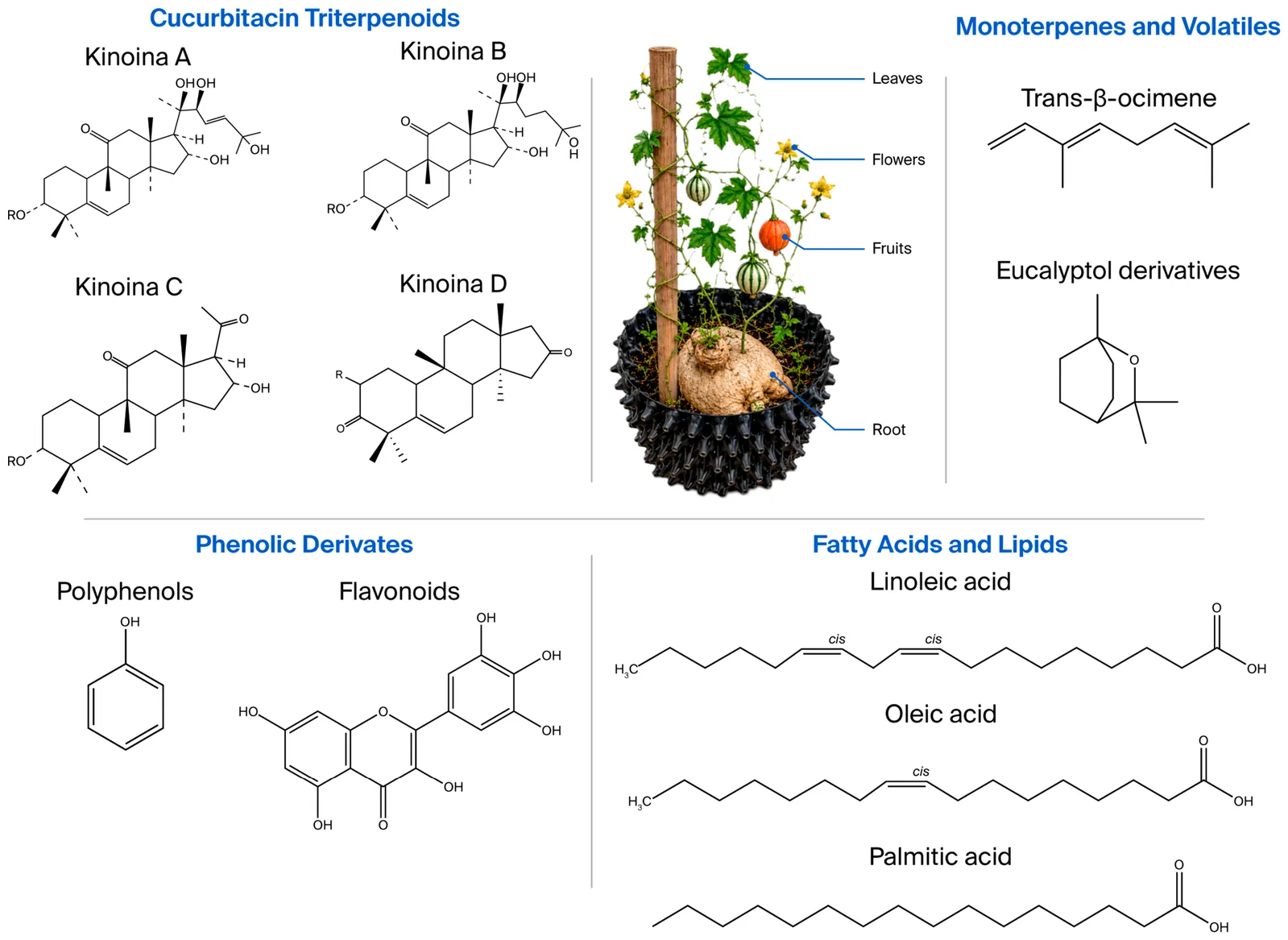
Morphological features of *Ibervillea sonorae* and representative chemical structures of its primary bioactive phytochemical classes. Schematic illustration of *I. sonorae* highlighting key botanic structures (leaves, flowers, fruits, and tuberous root) alongside the chemical structures of identified secondary metabolites, including cucurbitacin-type triterpenoids (kinoins A, B, C, and D), phenolic derivatives (polyphenols and flavonoids), monoterpenes/volatiles (trans-β-ocimene and eucalyptol derivates), and major fatty acid constituents (linoleic, oleic, and palmitic acids). Chemical structures were adapted and redrawn based on structural data reported by Huerta-Reyes et al. [5,8,9,10,11].

*Ibervillea sonorae* (S. Watson) Greene, commonly known as “wereke”, is a perennial species of the Cucurbitaceae family native to northern Mexico. The vernacular name “wereke” (also spelled “wareke”, “wareque”, or “wareki”) is associated with the traditional knowledge of Indigenous communities in northwestern Mexico. In particular, “wareki” has been documented among the Mayo people, while the plant is known by different vernacular names among other Indigenous groups, including “Hantyax” among the Comcaac (Seri) and “Uu kau chaani” among the Yaqui [4,5]. Although the term “wareki” is specifically associated with the Mayo people, a definitive linguistic etymology of “wereke/wareke” could not be established from the available ethnobotanical literature. The plant is characterized by a tuberous root, yellow flowers, and oval fruits (Figure 1) [6]. The phytochemical profile of *I. sonorae* comprises several classes of secondary metabolites, including cucurbitacin-type triterpenoids, fatty acids, phenolic compounds, and monoterpenes (Figure 1 and Table 1) [6] Among cucurbitacin-type triterpenoids, kinoins A, B, C, and D have been structurally characterized and reported as relevant constituents of the species. These compounds belong to a chemically diverse group of cucurbitacins that have attracted considerable interest because of their broad range of reported biological activities, including antioxidant, anti-inflammatory, antiproliferative, and metabolic effects [6,7]. In addition to kinoins, other reported constituents, including fatty acids, phenolic compounds, and monoterpenes, contribute to the chemical diversity of *I. sonorae* and may also participate in the biological effects associated with its preparations [6]. The available evidence does not establish that the antihyperglycemic activity of *I. sonorae* is mediated exclusively by kinoins or by any single constituent. The biological activity of plant preparations may instead reflect the combined contribution of multiple phytochemicals and their interactions. Therefore, the phytochemical profile provides an important framework for understanding the pharmacological potential of *I. sonorae*, whereas the mechanistic evidence evaluated in this systematic review should be interpreted primarily in relation to the extracts and preparations tested experimentally. Accordingly, the compounds presented in Figure 1 should be regarded as representative phytochemical constituents reported for *I. sonorae*, rather than as evidence that any particular compound or chemical class is solely responsible for its antihyperglycemic effects.

**Table 1:** Main constituents and reported biological activities of *Ibervillea sonorae*

Compound Class	Key Constituents	Biological Activity/Relevance	References
Cucurbitacin triterpenoids	kinoins A, B, C, and D	Antihyperglycemic potential, antioxidant activity, modulation of metabolic pathways	Huerta-Reyes et al. [5]; Delgado-Tiburcio et al. [7]
Monoterpenes and volatiles	Trans-β-ocimene, eucalyptol derivatives	Antimicrobial, synergistic antioxidant support	Torres-Moreno et al. [4]
Fatty acids and lipids	Linoleic, oleic, and palmitic acids	Membrane modulation, attenuation of metabolic inflammation	Torres-Moreno et al. [4]
Phenolic derivatives	Polyphenols, flavonoid traces	Free radical scavenging activity, inhibition of α-glucosidase/α-amylase	Torres-Moreno et al. [4]; Sota-Esparza et al. [12]

Data compiled form previously published preclinical studies.

Several in vitro and in vivo studies have reported promising antihyperglycemic effects of *I. sonorae*. Moreover, experimental studies have suggested that extracts and isolated compounds from *I. sonorae* may improve glucose homeostasis through multiple complementary mechanisms. These include activation of AMPK (adenosine monophosphate-activated protein kinase) and AKT (protein kinase) signaling pathways, enhancement of GLUT4 (Glucose Transporter Type 4) translocation and glucose uptake, modulation of insulin sensitivity, and inhibition of carbohydrate-digesting enzymes. In addition, the pleiotropic biological activities of cucurbitacins have generated interest in their potential applications beyond metabolic disorders, including cancer and inflammatory diseases. Nevertheless, the available evidence remains fragmented and has not yet been systematically synthesized. A comprehensive evaluation of the current literature is therefore needed to assess the strength and consistency of the evidence, identify the principal molecular mechanisms underlying its reported antihyperglycemic effects, evaluate methodological quality, and determine priorities for future research. Of note, systematic reviews play an essential role in integrating preclinical evidence, identifying methodological limitations and gaps, and guiding the development of future experimental and clinical research. Therefore, the present systematic review aimed to comprehensively synthesize and critically evaluate the available preclinical evidence regarding the antihyperglycemic effects of *I. sonorae*, with particular emphasis on the reported molecular mechanisms of action, methodological quality, and potential therapeutic relevance.

## 2. Results

### 2.1. Included Studies

**Figure 2:**
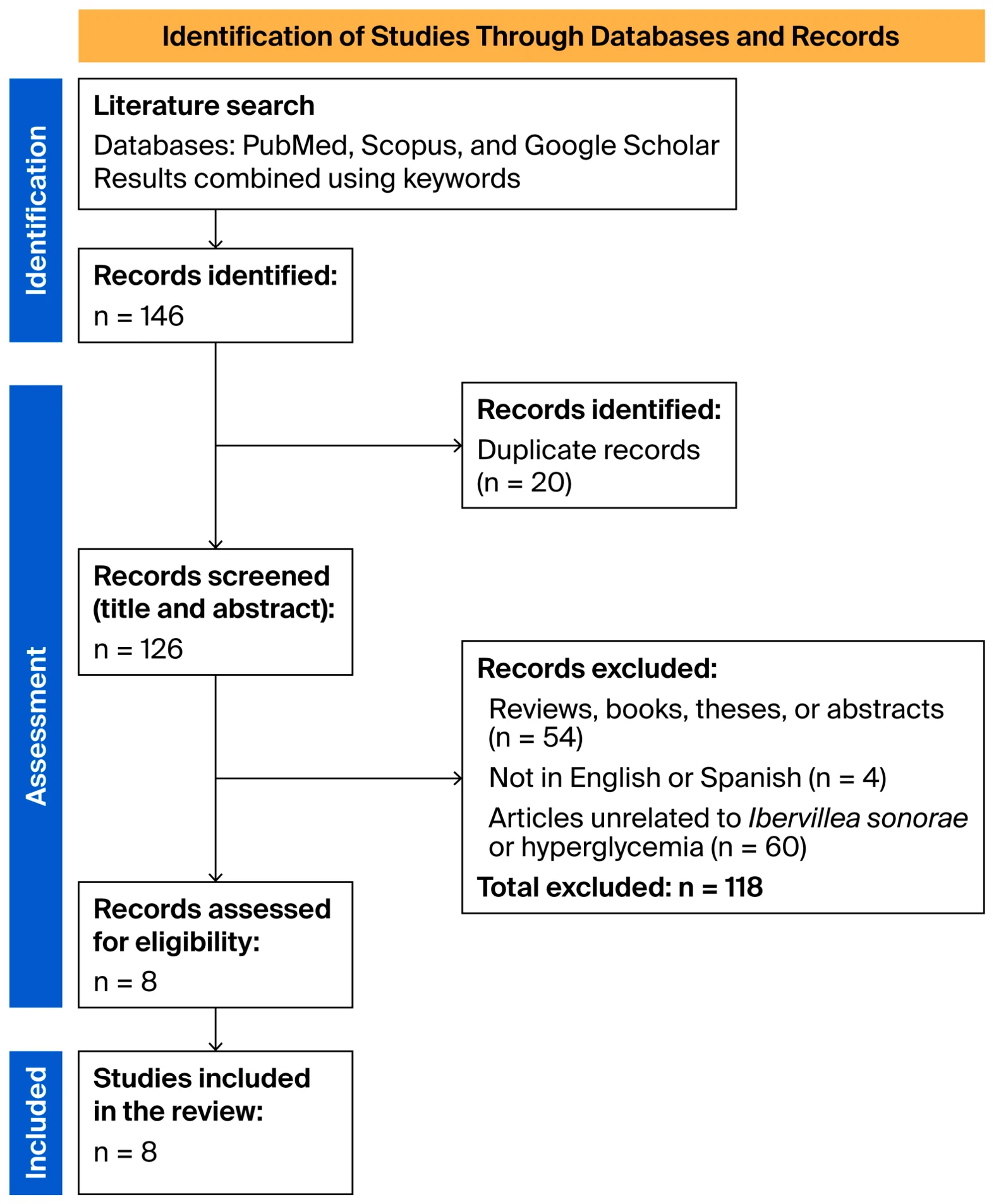
PRISMA 2020 flow diagram of the systematic literature search and study selection process. The flowchart illustrates the stages of identification, assessment, and inclusion of studies evaluating *I. sonorae* in relation to hyperglycemia [20].

The systematic search identified a total of 146 records in PubMed, Scopus, and Google Scholar. After removing 20 duplicates, 126 records were screened based on titles and abstracts. Of these, 54 were excluded because they were reviews (*n* = 26), theses (*n* = 13), abstracts (*n* = 5), and books (*n* = 10); 60 articles that did not address *I. sonorae* or hyperglycemia were also excluded, as well as 4 articles in a language other than Spanish or English. This left a total of eight articles for inclusion in this review. For the keywords used in the literature search, see Section 4.3. The complete process of identifying, selecting, determining eligibility, and including studies is summarized in the PRISMA 2020 flow diagram (Figure 2) [12].

### 2.2. Characteristics of Included Studies

Table 2 summarizes the included studies, which were exclusively conducted in Mexico and involved in vitro models, including the rat pancreatic β-cell line RIN-m5F, the murine preadipocyte cell lines 3T3-L1 and 3T3-F442A, and other in vivo models using Wistar rats and Institute for Cancer Research (ICR) or CD-1 mice [13,14,15,16,17,18,19,20]. Hyperglycemia in diabetes models was primarily induced by streptozotocin or high-calorie diets. Interventions included aqueous extracts, decoctions, lyophilized extracts, and other plant-derived preparations of *I. sonorae*, administered across a wide range of doses and treatment durations. The methodological quality of animal studies was assessed using the ARRIVE 2.0 guidelines (Table 3), while risk of bias was evaluated with the SYRCLE tool (Table 4). Overall, studies presented a low to unclear risk of bias, mainly due to insufficient reporting of randomization procedures, allocation concealment, and blinding.

**Table 2:** Summary of preclinical studies evaluating the antihyperglycemic effects of *Ibervillea sonorae*.

Author (Year)	Model	Hyperglycemia Induction	Intervention	Dose	Main Outcomes
Animal Studies					
Rivera-Ramirez et al. (2011) [13]	Male ICR mice	High-fat diet + fructose	Aqueous extract	100, 200 and 400 mg/kg	↓ blood glucose
Castellanos-Jiménez et al. (2022) [14]	Wistar rats	High-fat diet	Decoction	ad libitum	↓ glucose, ↓ HOMA-IR
Gómez-Guzmán et al. (2023) [15]	Wistar rats	STZ	Aqueous extract of cultured cells	50 mg/kg	↓ blood glucose, no toxicity
Sánchez-Velarde et al. (2015) [16]	Mice	STZ	Root extract	100–400 mg/kg	↑ insulin sensitivity
Cellular In Vitro					
Zapata-Bustos et al. (2014) [17]	3T3-F442A preadipocytes 3T3-L1 preadipocytes	—	Lyophilized extract	1, 10, 30 and 50 µg/mL	↑ 2-NBDG uptake, inhibited by insulin receptor and PI3K, AKT, and GLUT4 inhibitors in murine adipocytes; PI3K-independent uptake in human adipocytes
Semotiuk et al. (2020) [18]	RIN-m5F cells	—	Aqueous extract	0.1–10 µg/mL	↑ insulin secretion, α-glucosidase inhibition
Pérez-Ramírez et al. (2024) [19]	preadipocyte cells 3T3-L1	—	Aqueous extract	0.5 and 1.0 mg/mL in cells	↑ α-amylase inhibition ↑ glucose uptake in adipocytes
Enzyme Assay					
Sota-Esparza et al. (2024) [12]	α-glucosidase (enzyme assay)	—	Root extract	100–200 µg/mL	↑ α-glucosidase inhibition

HOMA-IR: homeostatic model assessment for insulin resistance; STZ: streptozotocin; AKT: protein kinase; PI3K: phosphatidylinositol 3-kinase; GLUT4: glucose transporter type 4. ↑: increase or enhancement; ↓: decrease or reduction.

**Table 3:** Risk and bias criteria in accordance with the ARRIVE 2.0 guidelines.

Study	Studio Design	Sample Size	Inclusion and Exclusion Criteria	Randomization	Blinding	Measurement Methods	Statistical Methods	Experimental Models	Experimental Procedures	Results
Rivera-Ramirez et al. (2011) [13]	+	+	−	−	−	+	+	+	+	+
Castellanos-Jiménez et al. (2022) [14]	+	+	+	+	−	+	+	+	+	+
Gómez-Guzmán et al. (2023) [15]	+	+	+	+	−	+	+	+	+	+
Pérez-Ramírez et al. (2024) [19]	+	+	+	+	−	+	+	+	+	+
Sánchez-Velarde et al. (2015) [16]	+	+	+	+	−	+	+	+	+	+

Risk of bias report: Yes (+) or no (−). ARRIVE 2.0 Guidelines for Reporting on Animal Research [21].

**Table 4:** Risk of bias assessment of in vivo studies evaluating *Ibervillea sonorae* using the SYRCLE tool.

Study	Random Sequence Generation	Allocation Concealment	Blinding	Incomplete Outcome Data	Selective Reporting	Overall Risk
Rivera-Ramirez et al. (2011) [19]	Unclear	Unclear	Unclear	Low	Low	Unclear
Castellanos-Jiménez et al. (2022) [14]	Low	Unclear	Unclear	Low	Low	Low
Gómez-Guzmán et al. (2023) [15]	Unclear	Unclear	Unclear	Low	Low	Unclear
Pérez-Ramírez et al. (2024) [19]	Low	Unclear	Unclear	Low	Low	Low
Sánchez-Velarde et al. (2015) [16]	Unclear	Unclear	Unclear	Low	Low	Unclear

### 2.3. Narrative Synthesis

In chemically induced, high-fat, or high-fructose diabetes models, the included studies generally reported antihyperglycemic effects following treatment with *I. sonorae* preparations. Despite variation in extraction methods, dosages, and treatment duration, the included studies reported significant reductions in fasting blood glucose levels compared with control groups in models of impaired glucose regulation. Improvements in glucose tolerance and insulin sensitivity indices were also reported in several experimental models, suggesting that the metabolic effects of *I. sonorae* may extend beyond a potential modulation of carbohydrate digestion and may involve changes in peripheral glucose handling and insulin responsiveness (Table 2). However, the extent to which these effects are attributable to specific molecular pathways remains incompletely characterized.

A recurring pattern in the mechanistic findings involves components of insulin-related signaling. Several studies reported changes in AKT phosphorylation and GLUT4 translocation in skeletal muscle and adipose tissue following treatment with *I. sonorae* preparations. These findings are consistent with enhanced insulin-dependent glucose uptake; however, the available evidence does not establish that *I. sonorae* directly activates the PI3K/AKT pathway or that changes in these signaling components are solely responsible for the observed antihyperglycemic effects. Similarly, changes involving AMPK signaling have been reported in association with metabolic improvements in some experimental models. Given the established role of AMPK in regulating cellular energy homeostasis and glucose uptake, these findings provide a plausible mechanistic framework for interpreting the metabolic effects of *I. sonorae*. Nevertheless, AMPK activation should be considered a potential mechanism rather than a consistently demonstrated or definitively established pathway across the included studies. Likewise, observations involving IRS-1 (Insulin receptor substrate 1) signaling in some models may be compatible with improved insulin signaling, but they do not by themselves establish a direct action of *I. sonorae* at the proximal insulin-signaling level.

In addition to these peripheral effects, several studies suggested a possible pancreatic contribution to the antihyperglycemic activity of *I. sonorae*. Histological assessments in streptozotocin-induced diabetes models indicated partial preservation of pancreatic β-cell architecture in some experimental settings, accompanied by increased circulating insulin levels. Although these findings may be consistent with insulinotropic or β-cell-protective effects, the underlying mechanisms have not been directly established. Thus, the available evidence supports the possibility that *I. sonorae* preparations may influence pancreatic function under conditions of experimental hyperglycemia, but whether this reflects direct cytoprotection, enhanced insulin secretion, or secondary metabolic effects remains to be determined.

At the intestinal level, in vitro enzyme assays demonstrated inhibition of α-glucosidase and α-amylase activities, providing direct evidence that *I. sonorae* preparations can interfere with carbohydrate-digesting enzymes under experimental conditions. This activity suggests a potential contribution to attenuating postprandial glucose excursions through delayed carbohydrate digestion. However, because these findings were obtained primarily from in vitro assays, their contribution to the overall antihyperglycemic effects observed in vivo cannot be established. Rather, intestinal enzyme inhibition may represent one component of a broader, potentially multimodal pharmacological profile involving carbohydrate digestion, peripheral glucose utilization, insulin signaling, and pancreatic function.

Despite these promising findings, substantial methodological heterogeneity was observed among the included studies. Variations in extraction procedures, including aqueous infusions, decoctions, freeze-dried extracts, and isolated fractions, as well as differences in dosage regimens and treatment duration, limit direct comparison across experimental studies. Most studies also used relatively small sample sizes, further limiting the generalizability and robustness of the findings. Future studies using larger sample sizes, standardized extraction procedures, well-characterized phytochemical profiles, and harmonized experimental protocols are therefore needed to confirm the reproducibility of the reported antihyperglycemic effects and to clarify their underlying mechanisms and safety.

Taken together, the available evidence supports the biological plausibility of *I. sonorae* as a potential source of antihyperglycemic activity, while indicating that its effects may involve multiple complementary biological processes. The convergence of findings related to intestinal carbohydrate digestion, pancreatic function, insulin-related signaling, and peripheral glucose utilization is compatible with a multimodal pharmacological profile. However, these mechanisms should be regarded as proposed or potentially contributing mechanisms rather than as definitively established pathways. This distinction is particularly important given the limited pharmacokinetic characterization, the heterogeneity of the preparations studied, and the lack of standardized quantitative phytochemical profiling. Moreover, because most studies evaluated complex plant extracts rather than isolated constituents, the contribution of individual phytochemicals, including kinoins, to these effects remains unresolved. Further studies incorporating standardized preparations, phytochemical quantification, pharmacokinetic characterization, selective pathway inhibitors, and genetic approaches are needed to establish causal relationships between specific constituents, molecular pathways, and the antihyperglycemic effects of *I. sonorae*.

## 3. Discussion

**Figure 3:**
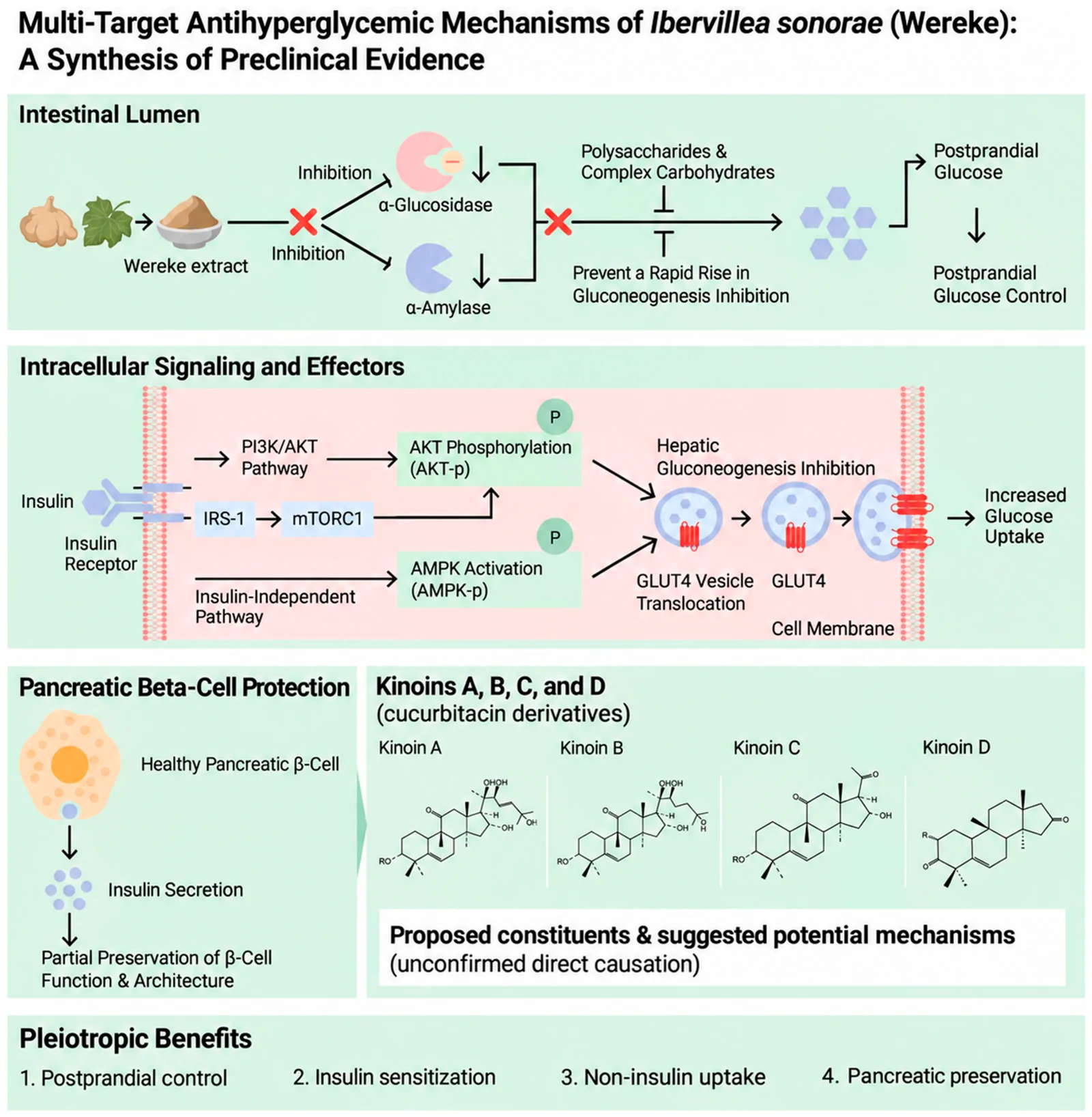
Integrated mechanistic framework of the reported antihyperglycemic effects of *Ibervillea sonorae*. The diagram integrates preclinical evidence regarding *I. sonorae* preparations, representative phytochemical constituents, molecular targets, biological effects, and potential antihyperglycemic relevance. The figure highlights the multimodal nature of the reported antihyperglycemic activity and identifies key mechanistic gaps relevant to future pharmacological and translational research.

Overall, the studies included in this systematic review demonstrated that *I. sonorae* reduced blood glucose levels, improved insulin sensitivity, and enhanced glucose uptake. The reported mechanisms include inhibition of carbohydrate-digesting enzymes and involvement of AKT-related signaling, whereas AMPK-associated signaling remains a potential mechanism requiring further validation (Figure 3). The results suggest a partially insulin-independent effect, with a possible preservation of pancreatic β cells. Further mechanistic refinement and pharmacological standardization are needed to determine whether these preclinical findings can be translated to clinical research.

### 3.1. AMPK as a Central Metabolic Regulator

AMPK is a central regulator of cellular energy homeostasis that integrates changes in cellular energy status with metabolic responses [22,23]. Its activation promotes catabolic processes that support ATP production while limiting energy-consuming anabolic pathways, and it can regulate glucose uptake and GLUT4 trafficking in metabolically active tissues [22,23]. These properties make AMPK a relevant molecular pathway when interpreting the antihyperglycemic effects reported for *I. sonorae*. Although the included studies demonstrated reduced blood glucose levels, improved insulin sensitivity, or increased glucose uptake following treatment with *I. sonorae* preparations [13,17], the available evidence summarized in this review does not establish AMPK activation as a directly measured mechanism across these studies. Nevertheless, AMPK represents a plausible pathway through which changes in cellular energy sensing could contribute to the metabolic effects observed with *I. sonorae* preparations. This possibility is particularly relevant because AMPK-mediated glucose uptake can complement insulin-dependent glucose transport and may therefore be functionally relevant in conditions characterized by impaired insulin signaling [22,23,24,25].

AMPK may also interact with downstream metabolic signaling, including the mechanistic target of the rapamycin (mTOR) pathway. AMPK can inhibit mammalian target of rapamycin complex 1 (mTORC1) signaling under conditions of cellular energy stress, thereby linking energy availability with anabolic metabolism [23,26]. Although direct measurements of mTOR were not reported in the included studies, this pathway may represent a potential downstream mechanism within the broader metabolic network illustrated in Figure 3. Therefore, the AMPK–mTOR relationship should be regarded as mechanistic context rather than direct evidence of pathway activation by *I. sonorae*. Importantly, the available evidence does not establish whether any specific phytochemical constituent of *I. sonorae*, including kinoins, directly modulates AMPK. The included studies evaluated extracts or other plant-derived preparations rather than isolated kinoins. Consequently, experimental studies using standardized extracts, isolated compounds, selective pathway inhibitors, or genetic approaches are needed to determine whether AMPK is directly involved in the antihyperglycemic effects of *I. sonorae* and to identify the upstream molecular events responsible for this potential response.

### 3.2. AKT Signaling and Insulin-Dependent Glucose Uptake

The insulin signaling pathway represents another important mechanism through which *I. sonorae* preparations may enhance glucose disposal. Insulin binding to its receptor activates a signaling cascade involving insulin receptor substrates and PI3K, ultimately promoting AKT activation and GLUT4 [24,25]. Impairment of this pathway is a major contributor to insulin resistance and reduced glucose disposal in metabolic disease [24,25].

Among the included studies, Zapata-Bustos et al. [17] provided the most direct evidence for the involvement of this signaling network. An aqueous extract of *I. sonorae* increased 2-NBDG uptake in murine and human adipocytes, including insulin-resistant cells. In murine adipocytes, pharmacological inhibition of the insulin receptor, PI3K, AKT, or GLUT4 blocked extract-induced glucose uptake, supporting the involvement of these components in the response [17]. However, this effect was not completely conserved in human adipocytes, in which inhibition of PI3K did not prevent extract-induced glucose uptake, suggesting that a PI3K-independent mechanism may contribute to the response in human cells [17].

The convergence between insulin-dependent and alternative glucose uptake pathways is particularly relevant to the interpretation of the antihyperglycemic activity of *I. sonorae*. The findings from murine adipocytes indicate that the insulin receptor–PI3K–AKT–GLUT4 axis can participate in the response to the extract, whereas the results obtained in human adipocytes indicate that glucose uptake may also occur through a PI3K-independent pathway [17]. Thus, the available evidence does not support a single uniform mechanism but rather suggests that the metabolic effects of *I. sonorae* may involve multiple signaling routes that vary according to cellular context. This potential convergence is illustrated in Figure 3.

Importantly, these findings were obtained using a plant extract rather than isolated phytochemicals. Therefore, the involvement of the insulin receptor, AKT, GLUT4, or alternative signaling pathways cannot be attributed to a specific constituent such as kinoins. Further studies using chemically standardized extracts and isolated compounds, together with pathway-specific inhibitors or genetic approaches, are required to determine which constituents are responsible for these effects and whether the same mechanisms operate in vivo.

### 3.3. Intestinal Enzyme Inhibition and Postprandial Control

The inhibition of carbohydrate-digesting enzymes represents an additional mechanism that may contribute to the antihyperglycemic activity of *I. sonorae*. α-Amylase and α-glucosidase participate sequentially in the digestion of dietary carbohydrates, and their inhibition can reduce the rate of glucose release and consequently attenuate postprandial increases in blood glucose [27]. The studies included in this review demonstrated that *I. sonorae* preparations inhibited α-glucosidase activity in enzyme-based and cellular experimental systems [12,18], and α-amylase inhibition was also reported for an aqueous extract in an in vitro model [19]. These findings suggest that, in addition to mechanisms involving peripheral glucose uptake and insulin-related signaling, *I. sonorae* may influence glycemic regulation by modulating carbohydrate-digesting enzyme activity. However, the available evidence remains heterogeneous with respect to extract type, concentration, and experimental model, precluding direct comparison of the magnitude of enzyme inhibition across studies.

The coexistence of effects on carbohydrate digestion, glucose uptake, and insulin-related responses suggests that the antihyperglycemic activity of *I. sonorae* may involve multiple complementary mechanisms instead of a single pharmacological pathway. Such multi-target activity is frequently observed with medicinal plant preparations containing chemically diverse bioactive constituents [3,5]. Nevertheless, the identification of potentially complementary mechanisms should not be interpreted as evidence that each molecular pathway is directly regulated by a specific phytochemical constituent.

Although the phytochemical composition of *I. sonorae* has been partially characterized, including the identification of cucurbitacin derivatives such as Kinoins [5,7], the mechanistic findings summarized in this review should be interpreted with caution. Most of the included studies evaluated crude extracts, aqueous preparations, decoctions, or other plant-derived preparations rather than isolated phytochemical constituents [12,13,14,15,16,17,18,19]. Consequently, the observed effects on glucose uptake, insulin-related signaling, carbohydrate-digesting enzymes, and glycemic control should primarily be attributed to the biological activity of the tested preparations as a whole, but not to the individual compounds. Synergistic or additive interactions among multiple phytochemicals may also contribute to the observed biological effects [3,5,7]. Therefore, although kinoins represent promising bioactive constituents based on the existing phytochemical evidence, their specific contribution to the antihyperglycemic mechanisms of *I. sonorae* remains to be established. Future studies using purified compounds, chemically standardized extracts, comparative pharmacological approaches, and target-specific experimental models are needed to determine whether *I. sonorae* individual constituents act on specific molecular pathways or whether the observed activity results predominantly from interactions among multiple constituents.

### 3.4. Potential Involvement of Inflammatory Signaling

Chronic low-grade inflammation represents an additional pathophysiological factor that may influence the metabolic pathways discussed above. In type 2 diabetes, persistent inflammatory and oxidative stress responses can impair insulin signaling through mechanisms involving inhibitory serine phosphorylation of IRS-1 and activation of inflammatory pathways such as nuclear factor kappa B (NF-κB), thereby contributing to insulin resistance and impaired glucose disposal [28,29]. Thus, modulation of inflammatory signaling could potentially complement the metabolic effects of *I. sonorae*, including those related to insulin signaling and glucose uptake. However, inflammatory biomarkers and signaling pathways were not systematically evaluated in the included studies. Evidence from studies outside of the antihyperglycemic models included in this review provides some biological context for this possibility. *I. sonorae* extracts have demonstrated anti-inflammatory and antioxidant properties [4], while cucurbitacins have been reported to modulate inflammatory signaling pathways, including Janus kinase/signal transducers and activators of transcription (JAK/STAT) and NF-κB [7]. These observations provide biological support for the possibility that the plant and some of its phytochemical constituents may exert effects beyond direct glucose regulation. Nevertheless, these findings should be interpreted as supportive mechanistic context for how anti-inflammatory activity mediates the antihyperglycemic effects of *I. sonorae*. Thus, future studies should integrate inflammatory biomarkers and pathway-specific measurements, including NF-κB, JAK/STAT, and inflammatory cytokines, with metabolic endpoints to determine whether anti-inflammatory activity contributes to the glucose-lowering effects of *I. sonorae*.

### 3.5. Safety Considerations

Safety represents an important consideration when evaluating the therapeutic potential of *I. sonorae*, particularly because the plant contains cucurbitacin-derived compounds, some of which have been associated with potent biological activities and cytotoxic effects at higher concentrations [7]. Among the included in vivo studies, Gómez-Guzmán et al. [15] reported no evidence of toxicity under the experimental conditions evaluated. The remaining in vivo studies primarily focused on glycemic and metabolic outcomes without systematic toxicity assessment. Therefore, the available evidence provides only preliminary information regarding the tolerability of *I. sonorae* preparations and should not be interpreted as evidence of long-term safety. The available studies were limited in treatment duration and did not systematically assess chronic toxicity, pharmacokinetics, or comprehensive dose–response relationships.

An additional safety consideration regarding *I. sonorae* is the chemical variability of plant-derived preparations. Differences in extraction procedures, plant material, preparation methods, and concentrations of bioactive constituents may substantially influence both pharmacological activity and toxicity. This issue is particularly relevant for *I. sonorae*, because most of the included studies evaluated crude extracts, aqueous preparations, or decoctions rather than chemically standardized preparations. Consequently, the safety profile observed with one experimental preparation cannot be extrapolated to other extracts with different phytochemical compositions. Future studies should therefore incorporate standardized phytochemical characterization, extended toxicity assessment, pharmacokinetic profiling, and systematic dose–response analyses to establish a more reliable therapeutic window for *I. sonorae* preparations.

### 3.6. Methodological Heterogeneity and Translational Limitations

Several limitations should be acknowledged when interpreting the findings of this systematic review. First, the number of available studies evaluating the antihyperglycemic effects of *I. sonorae* remains relatively limited, reflecting the still-emerging nature of research on this medicinal plant and restricting the overall breadth of evidence available for analysis. In addition, considerable methodological heterogeneity was observed among the included studies, including differences in diabetes induction models, extract preparation methods, dosing regimens, and treatment duration. Such variability limits direct comparability between studies and precludes the possibility of performing a quantitative meta-analysis.

The assessment of reporting quality and risk of bias provides additional context for interpreting preclinical evidence. According to the ARRIVE 2.0 assessment, most of the included studies showed adequate reporting across key domains, including study design, measurement methods, statistical methods, experimental models, experimental procedures, and results. However, reporting of randomization was consistently insufficient, while sample size and inclusion and exclusion criteria were incompletely reported in one study [30]. The SYRCLE assessment identified a low overall risk of bias in two studies (Castellanos-Jiménez et al., 2022, and Pérez-Ramírez et al., 2024) [14,19], whereas three studies (Rivera-Ramírez et al., 2011; Gómez-Guzmán et al., 2023; and Sánchez-Velarde et al., 2015) [13,15,16] were classified as having an unclear overall risk of bias. The main source of uncertainty was insufficient reporting of random sequence generation, while allocation concealment and blinding were assessed as having a low risk of bias across the evaluated studies. Incomplete outcome data and selective reporting were classified as unclear in all studies [30]. These methodological and reporting limitations do not invalidate the observed antihyperglycemic effects, but they reduce the certainty with which the magnitude and reproducibility of these effects can be interpreted. Therefore, future animal studies should provide more comprehensive reporting of experimental design, randomization procedures, allocation concealment, blinding, and sample size considerations in accordance with established reporting and risk-of-bias frameworks.

Another important limitation is that the current body of evidence relies exclusively on preclinical studies. Although in vitro and animal models provide valuable mechanistic insights, they cannot fully capture the complexity of human metabolic regulation in diabetes. In addition, variability in the phytochemical characterization of *I. sonorae* extracts across studies, largely due to differences in extraction methods and plant preparation, may lead to distinct phytochemical profiles and consequently influence the reported biological activity. This variability also limits the extent to which mechanistic findings obtained with one preparation can be generalized to other *I. sonorae* preparations. Finally, the geographic concentration of available studies and the absence of well-designed clinical trials represent important gaps in the literature. Future research should therefore focus on standardized extraction and phytochemical characterization, rigorous experimental design and reporting, expanded mechanistic investigations, and well-controlled clinical studies to more clearly establish the therapeutic potential, reproducibility, and safety profile of *I. sonorae* in the management of hyperglycemia.

## 4. Materials and Methods

### 4.1. Study Design and Protocol

This systematic review was conducted in accordance with the PRISMA 2020 guidelines [20]. The methodological protocol was defined a priori but was not registered in PROSPERO due to the exclusively preclinical nature of the included studies [31].

### 4.2. Research Question (PICO)

- Population: Cellular and animal models with diabetes mellitus.
- Intervention: Extracts or isolated compounds from *Ibervillea sonorae*.
- Comparison: Placebo, no treatment, or standard antidiabetic drugs.
- Outcomes: Changes in blood glucose levels and associated molecular mechanisms.

### 4.3. Information Sources and Search Strategy

A systematic search was conducted in PubMed, Scopus, and Google Scholar. MeSH terms, Boolean operators, and related keywords such as *Ibervillea sonorae*, wereke, hyperglycemia, glucose metabolism, and insulin resistance were used. The electronic searches were last conducted on 9 February 2026. Studies published between January 2010 and February 2026 in English or Spanish were considered, and in Google Scholar, the first results were filtered and sorted by relevance. Complete search strategies for each database are provided in Table 5 to ensure reproducibility.

### 4.4. Eligibility Criteria

Original in vitro and in vivo studies that evaluated the effects of *Ibervillea sonorae* on glycemic outcomes were included. Reviews, theses, books, studies without a control group, studies that did not report glucose-related outcomes, and publications in languages other than English or Spanish were excluded.

### 4.5. Study Selection

Two independent reviewers screened the titles and abstracts and then conducted a full-text assessment. Discrepancies were resolved by consensus.

### 4.6. Data Extraction

The following data were extracted: author, year, experimental model, induction of diabetes, intervention dose, and main results.

### 4.7. Quality Assessment and Risk of Bias

Methodological quality was assessed according to the ARRIVE 2.0 guidelines in animal models [21]. Likewise, the risk of bias in animal studies was assessed using the SYRCLE tool and classified as low, high, or uncertain risk [30].

### 4.8. Data Synthesis

Due to the considerable methodological heterogeneity among the included studies, a meta-analysis was not performed. The results were summarized narratively.

## 5. Conclusions

The present systematic review provides a comprehensive synthesis of the available preclinical evidence regarding the antihyperglycemic effects of *I. sonorae*. The analyzed studies consistently suggest that preparations derived from this medicinal plant may exert glucose-lowering effects through multiple complementary mechanisms, including enhanced glucose uptake, participation of insulin-related signaling pathways involving AKT and GLUT4, and inhibition of carbohydrate-digesting enzymes. AMPK-related mechanisms remain biologically plausible; however, their direct involvement has not been consistently established in the studies included in this review. Overall, the available findings highlight the potential of *I. sonorae* as a promising source of bioactive compounds for antihyperglycemic research. Nevertheless, the current evidence remains exclusively preclinical and is limited by methodological heterogeneity, variability in plant preparations, and insufficient phytochemical standardization. Further studies using chemically characterized and standardized preparations, rigorous mechanistic approaches, comprehensive safety assessments, and ultimately well-designed clinical trials are required before the therapeutic potential of *I. sonorae* can be established.

## 6. Future Directions

The present systematic review highlights the considerable therapeutic potential of *I. sonorae* as a source of bioactive compounds with antihyperglycemic activity. Nevertheless, several important knowledge gaps remain that should be addressed before its translation into clinical practice.

One of the main priorities is the phytochemical standardization of *I. sonorae* extracts. The studies included in this review employed different extraction procedures, plant materials, and preparation methods, resulting in substantial variability in phytochemical composition and biological activity. Future investigations should incorporate comprehensive phytochemical characterization using validated analytical techniques and establish standardized preparations with defined concentrations of the principal bioactive compounds, particularly cucurbitacins. Such standardization is essential to improve reproducibility, facilitate comparisons among studies, and ensure consistent pharmacological effects [6,7].

Another important research priority is clarifying the molecular mechanisms underlying the antihyperglycemic effects of *I. sonorae*. Although the available evidence supports the involvement of AKT signaling and suggests AMPK-related mechanisms, these pathways have only been evaluated in a limited number of experimental models. Future studies should investigate the interaction between insulin-dependent and -independent signaling pathways, as well as their crosstalk with inflammatory and oxidative stress pathways, including NF-κB, JAK/STAT, mTOR, and mitochondrial metabolic regulation. Integrating transcriptomic, proteomic, and metabolomic approaches could provide a more comprehensive understanding of the molecular targets responsible for the biological activity of this medicinal plant [7,22,29,32].

The safety profile of *I. sonorae* also requires further investigation. Although the available animal studies did not report significant toxicity, current evidence is limited to relatively short experimental periods and heterogeneous dosing protocols. Long-term toxicological studies, pharmacokinetic characterization, bioavailability analyses, and dose response evaluations are necessary to establish safe therapeutic ranges and identify potential adverse effects associated with prolonged administration of cucurbitacin-containing preparations [7].

Future experimental studies should also adopt more rigorous methodological standards. Greater adherence to the ARRIVE 2.0 guidelines, appropriate randomization procedures, allocation concealment, blinding, and adequate sample size calculations would substantially improve the quality and reproducibility of preclinical evidence [21,30]. Likewise, the use of standardized diabetic animal models and harmonized outcome measures would facilitate direct comparisons across studies and may eventually allow quantitative meta-analyses.

Finally, the complete absence of clinical evidence represents the most important barrier to the therapeutic translation of *I. sonorae*. Once standardized extracts and sufficient preclinical safety data become available, well-designed Phase I clinical trials should be conducted to evaluate safety, tolerability, pharmacokinetics, and optimal dosing in humans. Subsequently, randomized controlled clinical trials will be required to determine the efficacy of *I. sonorae* as a complementary therapeutic strategy for patients with impaired glucose metabolism or type 2 diabetes mellitus. Addressing these challenges will not only strengthen the scientific evidence supporting the traditional use of *I. sonorae* but may also contribute to the development of novel plant-derived therapeutic agents for metabolic diseases.

## Figures and Tables

**Table 5:** Search strategy adapted to each of the databases.

Database	Specific Search	Retrieved Studies
PubMed	(“*Ibervillea sonorae*” [All Fields] OR “wereke” [All Fields]) AND (“Hyperglycemia” [MeSH Terms] OR “Glucose Metabolism Disorders” [MeSH Terms] OR “Insulin Resistance” [MeSH Terms])	3
Scopus	(TITLE-ABS-KEY (*Ibervillea sonorae* OR wereke) AND TITLE-ABS-KEY (“hyperglycemia” OR “glucose metabolism” OR “insulin resistance”))	4
Google Scholar	(“*Ibervillea sonorae*” OR “wereke”) AND (“Hyperglycemia” OR “Glucose Metabolism” OR “Insulin Resistance”)	139

## Data Availability

No new data were created or analyzed in this study. Data sharing is not applicable to this article.

## Abbreviations

2-NBDG	2-(N-(7-Nitrobenz-2-oxa-1,3-diazol-4-yl) Amino)-2-Deoxyglucose
AKT	Protein Kinase B
AMPK	Adenosine Monophosphate-Activated Protein Kinase
ARRIVE	Animal Research: Reporting of In Vivo Experiments
GLUT4	Glucose Transporter Type 4
HOMA-IR	Homeostatic Model Assessment for Insulin Resistance
IRS-1	Insulin Receptor Substrate 1
JAK	Janus Kinase
MeSH	Medical Subject Headings
mTOR	Mammalian Target of Rapamycin
NF-κB	Nuclear Factor Kappa B
PICO	Population, Intervention, Comparison, Outcome
PI3K	Phosphatidylinositol 3-Kinase
PRISMA	Preferred Reporting Items for Systematic Reviews and Meta-Analyses
PROSPERO	International Prospective Register of Systematic Reviews
STZ	Streptozotocin
SYRCLE	Systematic Review Centre for Laboratory Animal Experimentation
